# Fetal surgery for open spina bifida

**DOI:** 10.1111/tog.12603

**Published:** 2019-09-27

**Authors:** Adalina Sacco, Fred Ushakov, Dominic Thompson, Donald Peebles, Pranav Pandya, Paolo De Coppi, Ruwan Wimalasundera, George Attilakos, Anna Louise David, Jan Deprest

**Affiliations:** ^1^ Clinical Research Fellow, Fetal Medicine Unit Elizabeth Garrett Anderson Wing University College London Hospital NHS Foundation Trust 235 Euston Road London NW1 2BU UK; ^2^ Specialist in Fetal Medicine, Fetal Medicine Unit Elizabeth Garrett Anderson Wing University College London Hospital NHS Foundation Trust 235 Euston Road London NW1 2BU UK; ^3^ Consultant in Paediatric Neurosurgery Specialist Neonatal and Paediatric Surgery Great Ormond Street Hospital for Children NHS Foundation Trust Great Ormond Street London WC1N 3JH UK; ^4^ Professor of Fetal Medicine, Fetal Medicine Unit Elizabeth Garrett Anderson Wing University College London Hospital NHS Foundation Trust 235 Euston Road London NW1 2BU UK; ^5^ Consultant in Fetal Medicine, Fetal Medicine Unit Elizabeth Garrett Anderson Wing University College London Hospital NHS Foundation Trust 235 Euston Road London NW1 2BU UK; ^6^ Professor of Paediatric Surgery Specialist Neonatal and Paediatric Surgery Great Ormond Street Hospital for Children NHS Foundation Trust Great Ormond Street London WC1N 3JH UK; ^7^ Professor of Obstetrics and Gynaecology Clinical Department Obstetrics and Gynaecology University Hospitals Leuven Leuven Belgium

**Keywords:** fetal surgery, fetoscopy, myelomeningocele, prenatal, spina bifida

## Abstract

**Key content:**

- Spina bifida is a congenital neurological condition with lifelong physical and mental effects.
- Open fetal repair of the spinal lesion has been shown to improve hindbrain herniation, ventriculoperitoneal shunting, independent mobility and bladder outcomes for the child and, despite an increased risk of prematurity, does not seem to increase the risk of neurodevelopmental impairment.
- Open fetal surgery is associated with maternal morbidity.
- Surgery at our institution is offered and performed according to internationally agreed criteria and protocols.
- Further evidence regarding long‐term outcomes, fetoscopic repair and alternative techniques is awaited.

**Learning objectives:**

- To understand the clinical effects, potential prevention and prenatal diagnosis of spina bifida.
- To understand the rationale and evidence supporting the benefits and risks of fetal repair of open spina bifida.
- To understand the criteria defining those who are likely to benefit from fetal surgery.

**Ethical issues:**

- The concept of the fetus as a patient, and issues surrounding fetal death or the need for resuscitation during fetal surgery.
- The associated maternal morbidity in a procedure performed solely for the benefit of the fetus/child.
- The financial implications of new surgical treatments.

## Introduction

### Spina bifida

Spina bifida is a congenital central nervous system malformation caused by incomplete closure of the neural tube by 28 days of gestation, leading to a defect in the bony spine. The worldwide incidence of spina bifida is approximately 4.63 per 10 000 births. In the UK, 700–900 pregnancies per year are estimated to be affected.^1^

**Figure 1:**
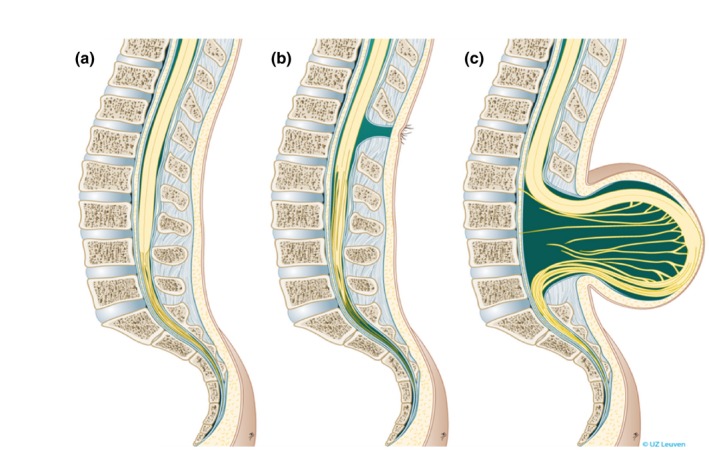
Types of spina bifida. (a) normal situation, (b) spina bifida occulta, (c) myelomeningocele. Reproduced with permission from Universitair Ziekenhuis Leuven, Belgium.

Spina bifida occulta (closed spina bifida) comprises a group of locoregional spinal malformations with intact overlying skin that are not associated with hydrocephalus or hindbrain herniation; the neurological implications are generally milder, and these conditions will not be considered further in this article. Spina bifida aperta (open spina bifida; Figure 1) involves exposure of the spinal cord and meninges through a defect in the overlying skin and bone. Two variations are recognised: myeloschisis, in which the neural tissue is ‘flush’ with the surrounding skin; and myelomeningocele (MMC), in which the spinal cord and meninges protrude beyond the plane of the skin as a cerebrospinal fluid (CSF)‐filled sac. These two variations will be collectively referred to as ‘spina bifida’ throughout.

Clinically, spina bifida leads to difficulties with mobility and ambulation, which is largely dependent on lesion level; by adulthood, independent ambulation is seen in 93% of patients with a sacral lesion, 91% with an L5 lesion, 57% with an L4 lesion and no patients with an L1–3 or thoracic lesion.^2^ Sensory deficits and orthopaedic abnormalities, such as talipes (clubfoot), kyphosis and scoliosis can also occur. Most patients with spina bifida experience neurogenic sphincter dysfunction resulting in impaired bladder and bowel control, which is commonly managed with a combination of toileting regimens, clean intermittent self‐catheterisation and medications such as laxatives, enemas and anticholinergics.^3^, ^4^ Sexual dysfunction may occur for several reasons, including erectile dysfunction for men, reduced genital sensation and psychological issues,^5^ although parenthood is possible. Brain changes are believed to develop in spina bifida as a result of leakage of CSF through the spinal lesion, causing hindbrain herniation (the Arnold–Chiari, or Chiari II, malformation). Impaired maturation of the CSF pathways is common and results in progressive cerebral ventricular dilation (hydrocephalus) in up to 80% of cases. Hydrocephalus is typically managed with a ventriculoperitoneal shunt or, in selected older children, endoscopic third ventriculostomy.^6^ Shunt complications, including failure and infection, may occur. Intelligence quotient (IQ) falls into the normal range for many adults with MMC, although the need for shunts and shunt complications are associated with a reduced IQ.^7^ Although not a lethal condition, spina bifida is associated with a reduced life expectancy and early mortality, particularly in those with higher lesions.^8^ Having a child with spina bifida is associated with higher levels of parental^9^ and sibling^10^ stress. When questioned, children and adolescents with spina bifida have lower self‐worth scores than their peers,^11^ but express positivity about their condition and hopefulness for their futures.^12^

### Prevention

Spina bifida is a multifactorial disease for which multiple underlying environmental, metabolic and genetic aetiologies have been proposed. An increased rate of spina bifida has been observed in women taking anticonvulsants^13^ and in women who have diabetes^14^ and/or are obese.^15^ The role of folic acid has been the most widely investigated. In 1991, the Medical Research Council (MRC) Vitamin Study^16^ demonstrated that pre‐ and post‐conception folic acid supplementation reduced the rate of open neural tube defects, including spina bifida, by approximately 70% in high‐risk women. The World Health Organization recommends supplementation with 400 μg folic acid daily from 2 months prior to conception. However, in the UK, only 31% of women take preconceptual folic acid, and this figure is even lower among younger and non‐Caucasian women.^17^ A 2017 study^18^ reported that mandatory folic acid fortification of wheat and/or maize flour had been implemented in 59 countries, preventing approximately 50 270 cases of spina bifida and anencephaly. Currently, the UK does not mandatorily fortify any food sources with folic acid, despite the suggestion that doing so in the 10 years following publication of the MRC evidence would have prevented over 2000 cases of neural tube defects.^19^ The UK Department of Health and Social Care launched a public consultation to consider the evidence, practicality and safety of mandatory folic acid food supplementation in early 2019.

### Diagnosis

**Figure 2:**
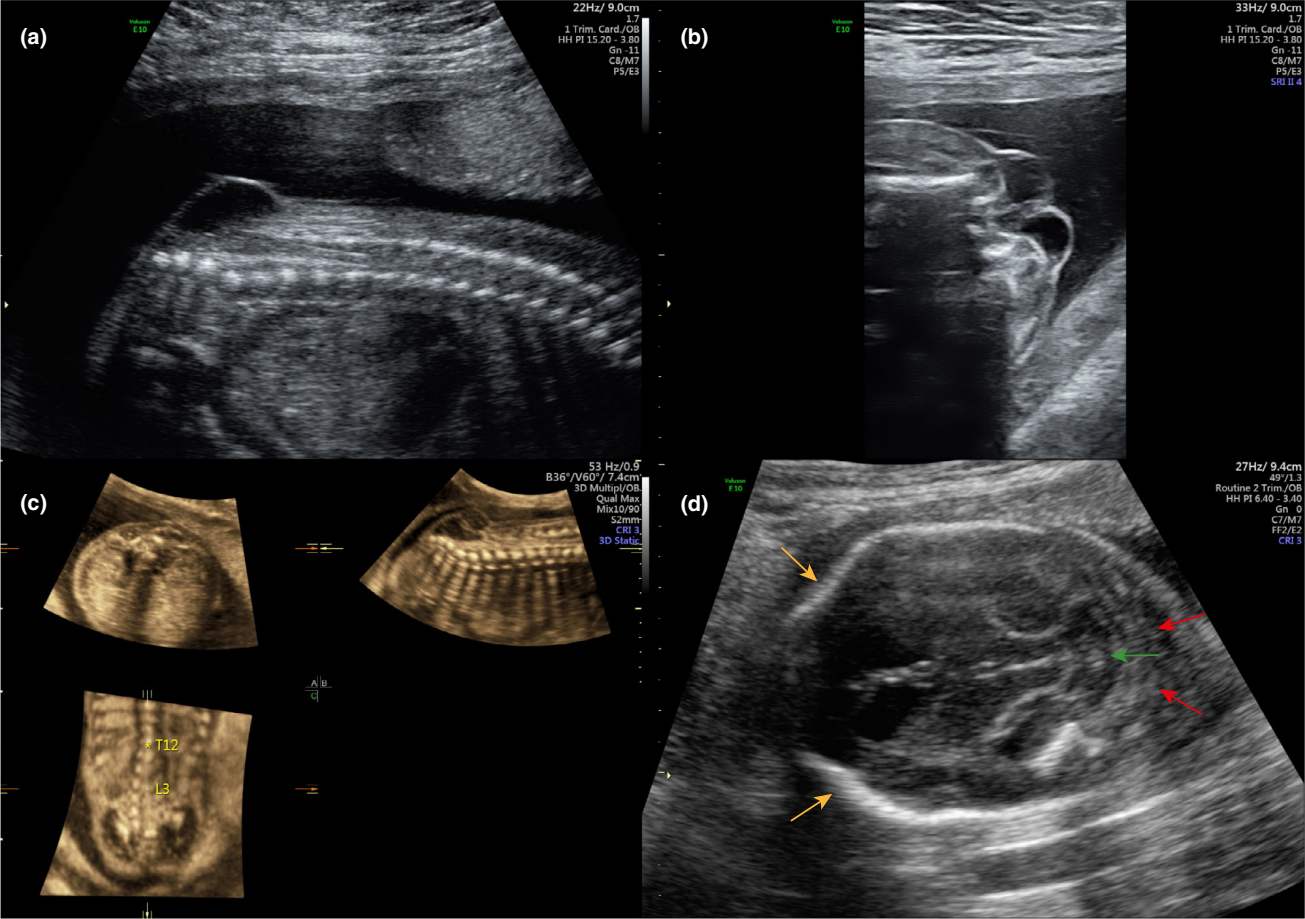
Antenatal diagnosis of spina bifida. Spinal lesion seen in (a) sagittal plane, (b) transverse plane and (c) 3D reconstruction. (d) Transverse section of fetal brain demonstrating ‘lemon’‐shaped skull (yellow arrows), ‘banana’‐shaped cerebellum (green arrow) and hindbrain herniation (red arrows). Reproduced with permission from University College London Hospitals.

Spina bifida aperta is usually detected in pregnancy during the second trimester (anomaly) ultrasound scan at 18^+0^ to 20^+6^ weeks of gestation (Figure 2). The typical intracranial characteristics include hindbrain herniation, a ‘lemon’‐shaped skull and a ‘banana’‐shaped cerebellum. The spinal lesion is commonly most easily visualised in the sagittal plane. The detection rate for spina bifida in populations that routinely offer second trimester ultrasound scans has been estimated at 68–100%.^20^, ^21^ In the UK, the Fetal Anomaly Screening Programme advises a minimum standard of 90% for detection of spina bifida.^22^ Given the high detection rate and widespread availability of second trimester ultrasound, screening for spina bifida by maternal serum alpha fetoprotein^23^ and confirmation of the diagnosis by amniocentesis^21^ are no longer routinely performed in many countries, including the UK.

Detection of spina bifida at an earlier gestation is beneficial for patient information and choice. It is also the case that termination of pregnancy, if ultimately chosen by the parents, is safer and more easily performed at earlier gestations. Several sonographic signs have been described to aid first trimester detection of spina bifida.^24^ The most extensively researched of these are the intracranial translucency, brainstem diameter, brainstem–occipital bone distance, aqueduct of Sylvius to occiput distance and frontomaxillary facial angle. Our own group recently described the use of a new sonographic marker, the ‘crash sign’,^25^ for diagnosing spina bifida in the first trimester. This, unlike other signs discussed, relies on pattern recognition without measurements. None of these signs have yet become well established in clinical practice.

### Management following antenatal diagnosis

Termination of pregnancy following an antenatal diagnosis of spina bifida is legal in many countries, including England, Wales and Scotland, without gestational age limit. A study in Belgium and Holland showed that 76% of patients chose this option,^26^ while in the UK, between 1991 and 2012, this figure was 81%.^19^

**Figure 3:**
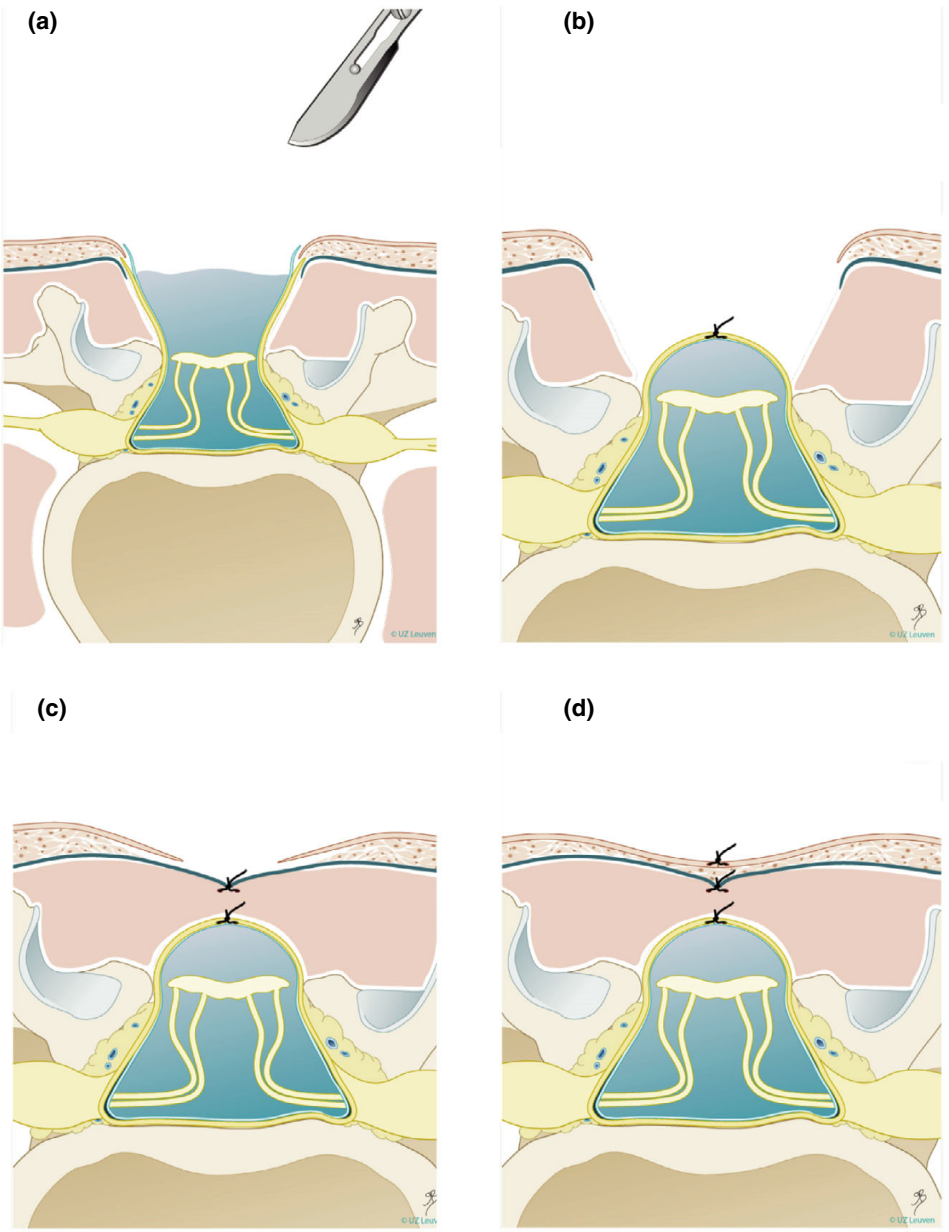
Open spina bifida repair technique. Step (a) shows untethering of the neural placode, followed by anatomical closure of the dura (b), myofascia (c) and skin (d). Reproduced with permission from Universitair Ziekenhuis Leuven, Belgium.

For continuing pregnancies, the standard treatment option is postnatal surgery to close the defect, protect the spinal cord and prevent ascending infection. This surgery is ideally performed within the first 48 hours of the baby's life. The standard multilayer repair (Figure 3) comprises untethering the spinal cord from the surrounding skin and meninges and resection of the myelomeningocele sac, followed by multilayer closure of the dural sac, lumbodorsal fascia and skin. Complex closures necessitating plastic surgery techniques are required in 20% of cases. Very often, women planning postnatal spina bifida repair give birth by caesarean section, although its benefit compared with vaginal delivery is not proven.^27^

## Open fetal repair of spina bifida

### Rationale

Both clinically^28^ and in animal models,^29^ the neurological effects of spina bifida have been shown to worsen throughout gestation. These observations led to the development of a ‘two‐hit’ hypothesis, in which the final neurological deficit results from a combination of the primary failure of neural tube formation and later injury from trauma and amniotic fluid toxicity. The corollary of this theory is, therefore, that earlier repair, while still in utero, should result in an improved outcome for the patient.

### Evidence

Animal models of spina bifida have suggested that open fetal repair is safe and effective.^30^ The first open fetal spina bifida repair in humans was performed in 1998^31^ and, over the following 5 years, several observational studies were published with generally encouraging results. In 2003, the Management of Myelomeningocele Study (MOMS)^32^ randomised clinical trial comparing outcomes after in utero and postnatal surgery was commenced. Three US centres already performing fetal spina bifida repair participated in the trial, while all other fetal medicine centres in the USA agreed not to perform the surgery while the trial was underway. Fetal surgery was offered in the trial between 19^+0^ and 25^+6^ weeks of gestation. A standardised method of repair was agreed across the three centres; this included a maternal laparotomy and stapled hysterotomy and layered neurosurgical repair as performed in postnatal surgery (Figure 3). Inclusion and exclusion criteria are shown in Table 1.

**Table 1:** Inclusion and exclusion criteria for fetal surgery in Management of Myelomeningocele Study (MOMS) trial^32^

Inclusion criteria	Exclusion criteria
Myelomeningocele (including myeloschisis) at level T1 through S1	Multifetal pregnancy
Maternal age ≥18 years	Insulin dependent pre‐gestational diabetes (since 2014, this is no longer a contraindication if well controlled)
Gestational age of 19^+0^ to 25^+6^ weeks of gestation*	Fetal anomaly not related to myelomeningocele
Normal karyotype	Kyphosis in the fetus of 30 degrees or more
	Current or planned cervical cerclage or documented history of cervical weakness
	Placenta praevia or placental abruption
	Short cervix (<20 mm) measured by transvaginal cervical ultrasound
	Obesity as defined by body mass index of 35 or greater (since 2014 this has been moved up to 40)
	Previous spontaneous singleton delivery prior to 37 weeks
	Maternal–fetal Rh isoimmunisation, Kell sensitisation or a history of neonatal alloimmune thrombocytopaenia
	Maternal HIV or hepatitis‐B status positive
	Known hepatitis‐C positivity; if unknown, screening is not required
	Uterine anomaly such as large or multiple fibroids or müllerian duct abnormality
	Previous surgery in the uterine corpus
	Patient does not have a support person (e.g. husband, partner, mother)
	Inability to comply with the travel and follow‐up requirements of the trial
	Patient does not meet other psychosocial criteria (as determined by the psychosocial interviewer using a standardised assessment) to handle the implications of the trial
	Maternal hypertension that would increase the risk of pre‐eclampsia or preterm delivery (including, but not limited to uncontrolled hypertension, chronic hypertension with end organ damage and new onset hypertension in current pregnancy)

* Criteria that have been amended since trial conclusion

The main outcomes from the MOMS trial (183 women)^33^, ^34^, ^35^ are shown in Table 2. Prenatal surgery significantly improved the rate of ventriculoperitoneal shunt placement (44% prenatal surgery versus 84% postnatal), hindbrain herniation at 12 months of age (64% prenatal versus 96% postnatal) and independent walking at 30 months of age (45% prenatal versus 24% postnatal). There was associated fetal morbidity, typically associated with prematurity and often with spontaneous membrane rupture (44%). Delivery occurred, on average, at 34 weeks of gestation in the prenatal surgery group and at 37 weeks of gestation in the postnatal group. Of note, 13% of the fetal surgery group delivered prior to 30 weeks of gestation. For the mother, risks included placental abruption (6%) and postoperative pulmonary oedema in 5%, which was at least partly attributed to the use of magnesium sulphate for tocolysis. Evaluation of the uterine scar at delivery showed an area of dehiscence in 9% of cases and full dehiscence in 2%; an increased rate of blood transfusion at delivery (9%) was also reported.

**Table 2:** Summary of main risks and benefits of prenatal surgery from the Management of Myelomeningocele Study (MOMS) trial^32^, ^33^, ^34^, ^35^

Outcome	Prenatal surgery, n (%)	Postnatal surgery, n (%)	*P* value
**Fetal benefits**			
Shunt placement at 12 months	40 (44)	77 (84)	<0.0001
Hindbrain herniation at 12 months	45 (64)	66 (96)	<0.001
Independent walking at 30 months	39 (44.8)	21 (23.9)	0.01
**Maternal risks**			
Pulmonary oedema	5 (5.5)	0 (0)	0.03
Placental abruption	6 (6.6)	0 (0)	0.01
Blood transfusion at delivery	8 (8.8)	1 (1.1)	0.02
Status of hysterotomy at delivery:			
Intact, well healed	57 (64.8)		
Very thin	21 (23.9)		
Area of dehiscence	8 (9.1)		
Complete dehiscence	2 (2.3)		
Spontaneous membrane rupture	40 (44.0)	7 (7.6)	<0.0001
Chorionic membrane separation	30 (33.0)	0 (0)	<0.0001
**Fetal/neonatal risks**			
Bradycardia during repair	8 (10)	0	0.003
Perinatal death	2 (3)	2 (2)	1.00
Average gestational age at birth	34.1+/− 3.1	37.3+/−1.1	<0.001
Gestational age at birth:			
<30 weeks	10 (13)	0	
30–34 weeks	26 (33)	4 (5)	
35–36 weeks	26 (33)	8 (10)	
≥37 weeks	16 (21)	68 (85)	
Average birthweight (g)	2383±688	3039±469	<0.001
Respiratory distress syndrome	16 (21)	5 (6)	0.008

In patients with a ventricle size <10 mm at assessment, 20% required ventriculoperitoneal shunt placement after prenatal surgery (versus 79% undergoing postnatal surgery). When the ventricle size was >15 mm at assessment, 79% of patients required shunt placement after prenatal surgery (versus 87% after postnatal surgery).

Following publication of this trial, further non‐randomised studies have reported similar short‐term outcomes. A review of cases from the Children's Hospital of Philadelphia in 2016^36^ showed that the risk of chorioamniotic membrane separation, preterm premature rupture of membranes (PPROM) and preterm birth were all increased when surgery was performed at the earlier gestational age range. Therefore, although the MOMS trial allowed entry from 19 weeks of gestation, it was recommended that fetal surgery should not be performed before 23 weeks of gestation.

### Long‐term outcomes

The long‐term outcomes of the MOMS trial have not yet been reported; therefore, cohorts operated prior to the MOMS trial are, at present, the only source of these data. Five‐year follow‐up studies of 30 fetal surgery cases performed prior to the MOMS trial have reported a shunt rate of 47–55%. Average or high‐average IQ scores were found in 90% of patients; this was significantly lower in those who had required a ventriculoperitoneal shunt compared with those who had not.^37^ Functional and self‐care scores were lower than for age‐matched population norms,^38^ but behavioural problems were no higher in fetal surgery patients than healthy controls.^39^

A 10‐year follow‐up of 42 fetal surgery cases^40^ reported that 79% of patients were ‘community ambulators’, 9% were ‘household ambulators’ and 14% were wheelchair‐dependent. Preschool ambulation was predictive of long‐term ambulation. ‘Normal bladder function’ (continence at all times) was reported in 26% of patients and normal bowel function in 31%; 74% of patients performed clean intermittent catheterisation. The overall rate of ventriculoperitoneal shunt placement was 43%, of which 61% had required at least one revision. No shunts had been inserted after 12 months of age. Most children could successfully complete everyday tasks at home and at school. When compared with population norms, abnormalities of these behavioural adaptive skills appeared to be more common than impairments in executive function. Non‐shunted children with normal early neurodevelopmental outcome were less likely to experience problems with executive function and behavioural adaptive skills.

A systematic review from our institution^41^ showed that, following open fetal surgery for spina bifida and other conditions, 48% of women had a subsequent pregnancy, with a preterm delivery rate of 20% and a uterine dehiscence/rupture rate of 7% and 11%, respectively.

### Ethical issues

#### The fetal patient

The concept of the fetus as a patient and a potential person independent of the mother is a source of ongoing scientific, societal, legal and philosophical debate. It is logical to infer that, when the pregnant woman decides to continue her pregnancy and asks for prenatal treatment, the fetus should be considered a patient, but that the pregnant woman should not be forced to undergo fetal treatment against her will.^42^ Fetal surgery for spina bifida has an associated risk of fetal death (3% in the MOMS trial), which is not statistically greater than the risk of neonatal death seen in those undergoing postnatal treatment (2%), but which is the direct result of an active choice by the parents rather than a natural consequence of disease progression. As fetal surgery is performed around the limits of fetal viability (23–26 weeks of gestation), decisions regarding the extent of resuscitation in the event of intra‐operative fetal distress and delivery are complex and should be discussed with parents before surgery.

#### Maternal morbidity

In line with accepted criteria, fetal surgery for spina bifida is almost exclusively offered to women who are themselves in good health. Fetal surgery offers no direct medical benefit to the mother; indeed, it poses risks to her not only during the procedure but also throughout the remainder of the index pregnancy and – potentially – during any future pregnancies. It is therefore crucial that maternal risks are minor and acceptable to the mother and her family. A full explanation of these risks must also be given to the patient in a non‐directive manner prior to her decision about whether or not to proceed with fetal surgery.^43^

#### Financial burden

Equitable distribution of limited resources within a pressurised system is a problem facing healthcare providers in many countries. The provision of fetal surgery for spina bifida may be seen as a new, expensive innovation that redirects funds from more ‘mundane’ scenarios. Within the UK, internal data from our own institution has found that the cost of an individual surgery is roughly equivalent to the standard tariff charge for postnatal surgery, although this does not include set‐up costs. In 2012, a US cost‐effectiveness evaluation^44^ used decision–analysis modelling and assumptions of improvement in clinical outcomes and risks based on the MOMS trial data. This study took into consideration the current fetal MMC patient, the mothers carrying the fetal MMC patient and possible future siblings of these patients. It found that fetal repair would save over $20,000 USD and gain almost one quality‐adjusted life year per case performed.

### Availability

Following publication of the MOMS trial, the availability of open fetal surgery for spina bifida increased. At the time of writing there are 23 centres offering this treatment option worldwide, including six in Europe.^45^ In 2019, the NHS England Highly Specialised Services team will be commissioning two fetal surgery centres to provide open repair of fetal spina bifida.

## Open fetal repair of spina bifida – surgery

### Preoperative

**Figure 4:**
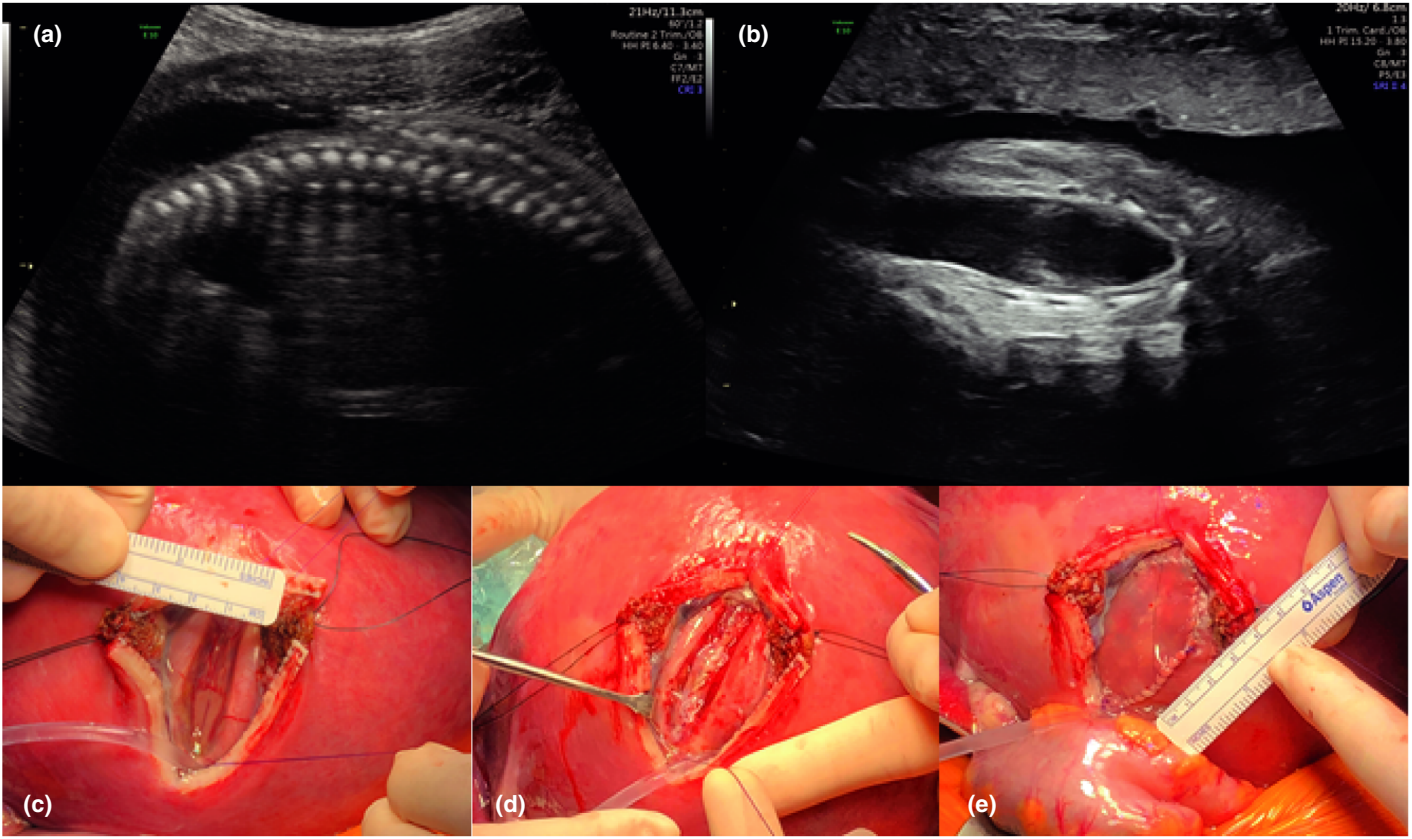
A case of myeloschisis. (a,b) Preoperative ultrasound findings of a relatively wide open lesion; (c) intraoperative images of the lesion prior to repair; (d) following closure of the dura and (e) application of a skin patch. Reproduced with permission from University College London Hospitals, London and Universitair Ziekenhuis Leuven, Belgium.

Most centres offering open fetal surgery for spina bifida use the MOMS trial criteria (Table 1) to determine eligibility for surgery; some also consider women with a body mass index of 40, those with well‐controlled insulin‐dependent diabetes or those who have previously undergone a lower segment caesarean section. A full, structural fetal ultrasound scan, including neurosonography and echocardiography, is performed to determine lesion level and size, the degree of any spinal kyphosis, the presence of hindbrain herniation and ventricular size and to exclude any additional abnormalities (Figures 3 and 4). Magnetic resonance imaging (MRI) is used as an adjunct to confirm intracranial findings and exclude any undetected abnormalities. As per the accepted criteria, amniocentesis is performed for karyotype or microarray. In line with the evidence discussed, surgery is typically planned to take place between 23^+0^ and 25^+6^ weeks of gestation.

### Intraoperative

**Figure 5:**
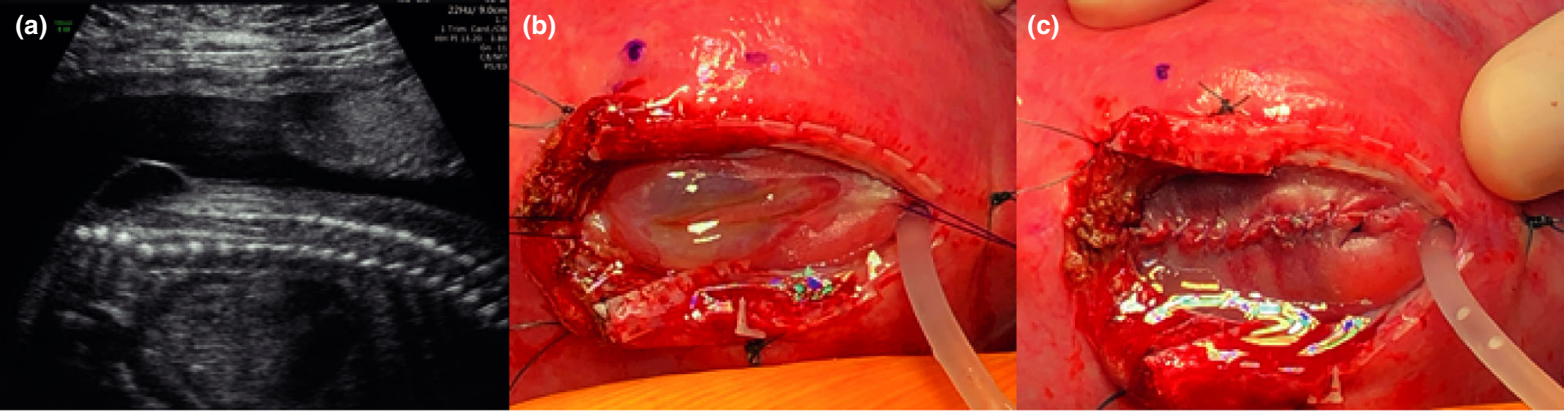
A case of myelomeningocele (same patient as Figure 2). (a) Preoperative findings of a covered lesion; (b) intraoperative images of the lesion prior to repair and (c) following skin closure. Reproduced with permission of University College London Hospitals and Universitair Ziekenhuis Leuven, Belgium.

An epidural is inserted prior to surgery to allow for postoperative pain management; deep inhalational general anaesthesia is used to reduce uterine contractility and tone. Maternal intravenous antibiotics are given and tocolysis is administered pre‐operatively and intra‐operatively. In North American centres, magnesium sulphate is commonly used for tocolysis, whereas in European centres, atosiban is usually preferred. The fetal heart rate is monitored throughout the procedure by ultrasound scanning. A transverse skin incision that is wider and higher than the Pfannenstiel incision is made, and the uterus is exposed. The placenta is mapped and the fetal position is adjusted to optimise access to the spina bifida lesion if required. A 6–8 cm stapled hysterotomy is made. Amniotic fluid is replaced throughout the procedure with warmed crystalloid fluid via a high flow rate infusion device. An intramuscular injection of analgesia and muscle relaxant is administered to the fetus. Fetal neurosurgical closure is performed as in postnatal surgery (Figures 3 and 5); in the event that a lesion is too wide to allow primary skin closure, a skin patch (e.g. Integra, Integra Life Sciences, New Jersey, USA) is applied and sutured to the fetal skin (Figure 4). The uterus is closed in two watertight layers: the first running, the second interrupted and inverting. Before the last few sutures of uterine closure, crystalloid fluid is allowed to accumulate and antibiotics are administered into the amniotic cavity. An omental flap is fixed over the suture line and the maternal laparotomy is closed in layers. A video recording of such a procedure is available as video [Link].

### Postoperative

**Figure 6:**
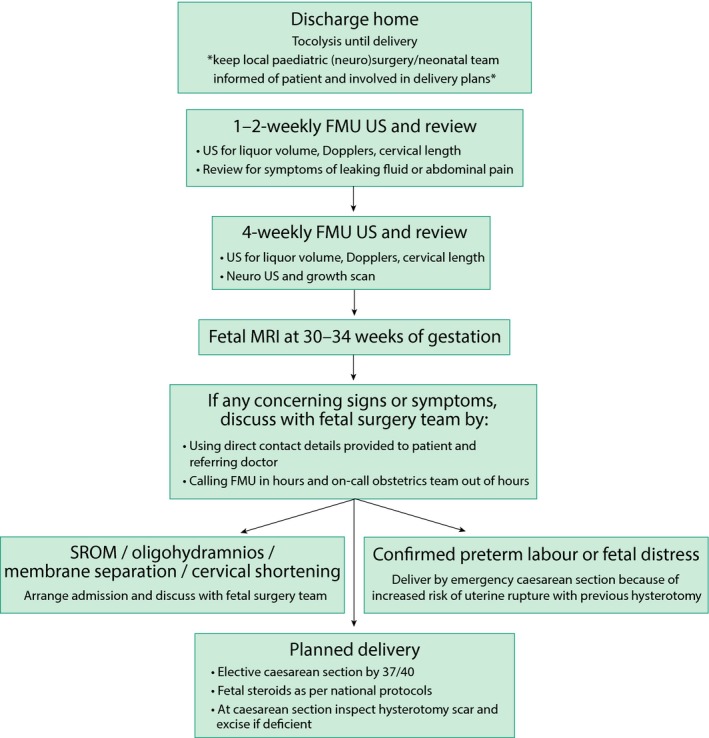
Outpatient postoperative pathway. FMU = fetal medicine unit; MRI = magnetic resonance imaging; SROM = spontaneous rupture of membranes; US = ultrasound scan

**Figure 7:**
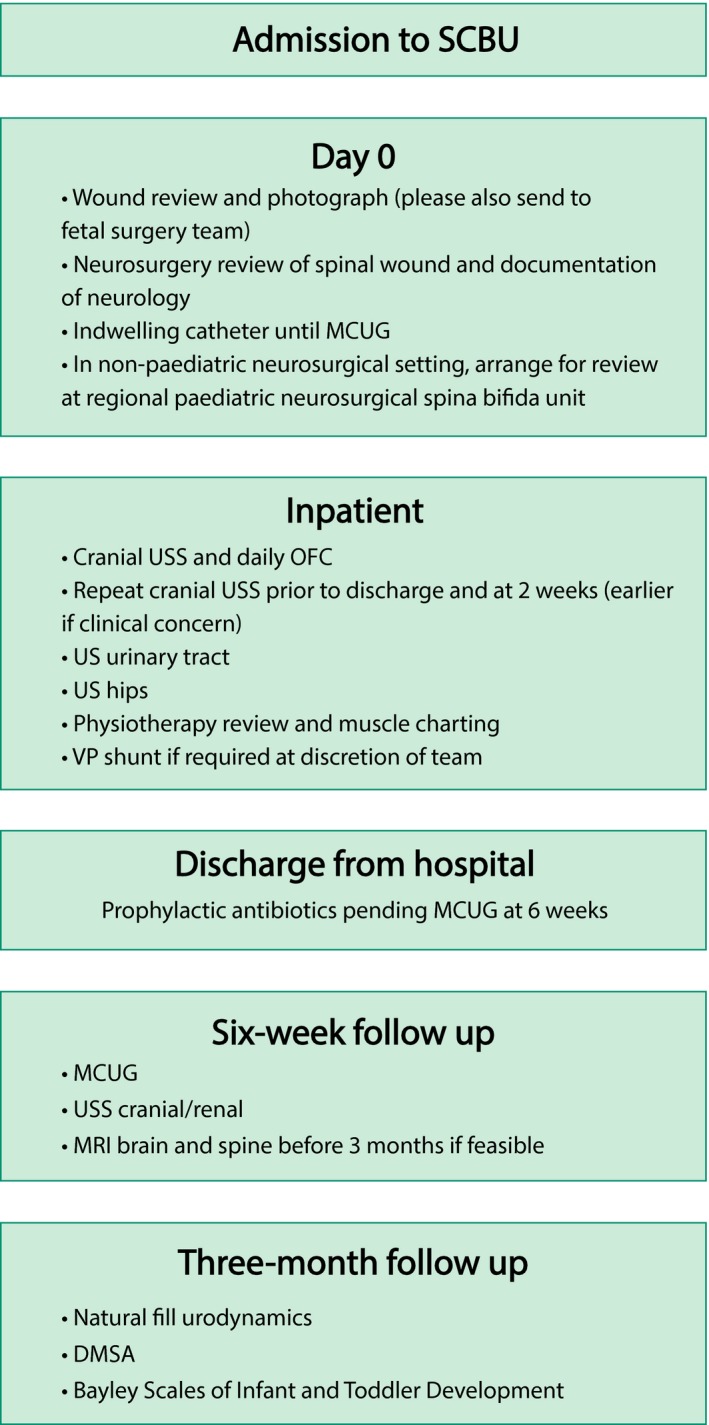
Neonatal protocol following open fetal surgery for spina bifida. DMSA = dimercaptosuccinic acid radionuclide scan; MCUG = micturating cystourethrogram; MRI = magnetic resonance imaging; OFC = occipito‐frontal circumference; SCBU = special care baby unit; USS = ultrasound scan; VP = ventriculoperitoneal

The patient is typically cared for in a high‐dependency setting for 24–48 hours, followed by an antenatal ward setting for 5–7 days. Initially, pain is managed by an epidural and later by oral analgesia. Tocolysis is continued and is usually taken for the rest of the pregnancy. Following discharge, the woman is advised to limit strenuous activities and to stop work. A protocol for follow up (Figure 6), involving regular ultrasound scans for cervical length and amniotic fluid levels, is followed and a further MRI scan is planned prior to delivery. If preterm labour occurs at any point following fetal surgery, then an emergency caesarean section must be performed because of the increased risk of uterine rupture with a recent hysterotomy. If preterm labour does not occur, then delivery is planned by elective caesarean section at 37 weeks of gestation. Caesarean delivery should be via a standard lower segment incision; it is recommended that the hysterotomy site is inspected and re‐sutured if deficient or dehiscent. Figure 7 shows a suggested protocol for neonatal care. Following open fetal surgery for spina bifida, a pregnancy interval of 2 years is recommended and all future deliveries should be by caesarean section.

## Future considerations

### Mini‐hysterotomy

A less invasive alternative to the surgical method described is the use of a ‘mini‐hysterotomy’, i.e. a uterine opening with a diameter of less than 4 cm, as opposed to the 6–8 cm opening commonly used.^46^ Through this opening, a standard multilayer microsurgical repair is performed. In a case series of 45 patients, there was a reduced PPROM rate (23%), a slightly higher gestational age at delivery (35 weeks) and a 95% intact hysterotomy site at delivery. Outcomes were only given until discharge from hospital, but as the same technique was used, one could hope that longer follow‐up will provide evidence of benefit for this technique.

### Fetoscopic repair of spina bifida

**Figure 8:**
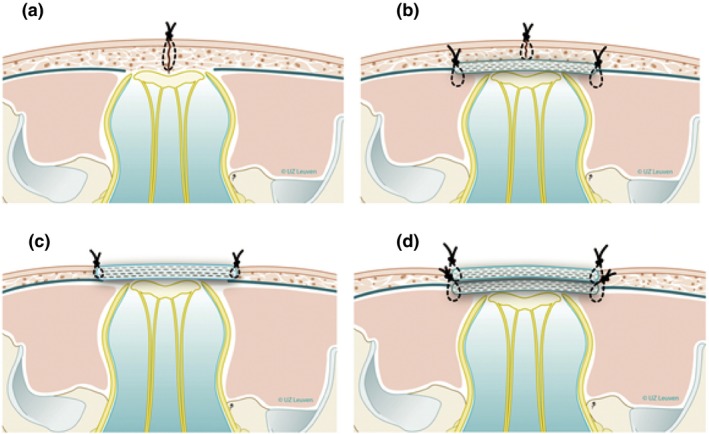
Examples of neurosurgical repair techniques reported in fetoscopic spina bifida repair. (a) Single layer repair (skin sutured); (b) double layer repair (subcutaneous patch and skin suture); (c) patch coverage; (d) double patch repair. Reproduced with permission of the Universitair Ziekenhuis Leuven, Belgium.

Alongside the development of open fetal surgery for spina bifida, there has been extensive interest in treating this condition antenatally via less invasive methods. Although the first fetal spina bifida repairs were via multiport fetoscopy, complications forced this to be temporarily abandoned.^47^ This was pursued in Germany and later in Brazilian and US centres. Unlike the standardised multilayer neurosurgical repair, which is based on the postnatal repair technique, there is no single agreed procedure for fetoscopic repair. Techniques vary in terms of maternal abdominal entry (fully percutaneous or laparotomy with exteriorised uterus), uterine entry (port numbers and sizes) and the neurosurgical technique itself (Figure 8). Fetoscopy is also controversial because, experimentally, uterine insufflation with CO_2_ causes fetal acidosis, yet the clinical relevance of that remains unknown.^48^

A systematic review of 11 fetoscopic spina bifida repair studies^47^ found no difference in the rate of ventriculoperitoneal shunting or hindbrain herniation reversal compared with data for open fetal surgery. It also found a higher preterm membrane rupture rate (79% versus 36%) and a higher need for additional neonatal treatment of the repair site (30% versus 7%). Rates of uterine dehiscence were lower in the fetoscopic group (0% versus 8%) and the rate of placental abruption did not differ between the two groups. A limitation is that the learning curve for this procedure is twice as long (up to 60 cases) as for spina bifida repair by hysterotomy.^49^

Therefore, currently, fetoscopic repair does not yet clearly confer a fetal benefit equal to open repair and appears to be associated with higher rates of prematurity and preterm rupture of membranes. However, techniques are heterogenous and some individual centres now report promising results. Given the potential for reduction in maternal morbidity and the possibility of vaginal delivery, it is hoped that further technical development and experience will result in an optimal technique to benefit the fetus and reduce harm to the mother. A 2017 American College of Obstetrics and Gynecology Committee Opinion^50^ recommended that, “at this time, fetoscopic fetal myelomeningocele repair cannot be recommended outside of an institutional review board‐approved investigational setting.”

### Instrument development

Our research teams have been developing this technology and instruments for use in fetal surgery. Currently, they are training on high‐fidelity in vivo models, exploring the extent to which a layered, watertight neurosurgical repair can be performed. The GIFT‐Surg project,^51^ with funding from the Wellcome Trust and Engineering and Physical Sciences Research Council (EPSRC), are working on a single‐orifice miniature access robot for endoscopic closure of spina bifida lesions.

## Conclusion

Spina bifida is a congenital central nervous system malformation with lifelong physical and mental effects. Open fetal repair of the spinal lesion has been shown to improve short‐term outcomes for the child, with the consequent risks of prematurity and maternal morbidity. Surgery in the UK is offered and performed according to internationally agreed criteria and protocols. Further evidence regarding long‐term outcomes, fetoscopic repair and alternative techniques is awaited.

### Disclosure of interests

The University College London Hospital (UCLH)/Great Ormond Street Hospital (GOSH) Centre for Prenatal Therapy is funded by the UCLH Charity and the GOSH Children's Charity. Our research on fetal surgery is, in part, funded by the Wellcome Trust (WT101957) and Engineering and Physical Sciences Research Council (ESPRC) (NS/A000027/1). DP and ALD are supported by the National Institute for Health Research University College London Hospitals Biomedical Research Centre, and PDC is supported by the National Institute for Health Research Great Ormond Street Hospital Biomedical Research Centre. JD is a member of the BJOG Editorial Board. PP is the Chair of the Fetal Anomaly Screening Programme Advisory Board. GA is an Associate Editor of *The Obstetrician & Gynaecologist*; he was excluded from editorial discussions regarding the paper and had no involvement in the decision to publish. GA is also the RCOG Council Representative for London: North, Central, East and North West. AS is Chair of the RCOG Trainees’ Committee.

### Contribution to authorship

AS proposed and wrote the article. JD researched, wrote and edited the article. All authors edited the article and read and approved the final version.

### Acknowledgements

Funding information: Wellcome Trust (WT101957); Engineering and Physical Sciences Research Council (ESPRC; NS/A000027/1).

## Supporting information


**Video S1.** Video of open fetal surgery for spina bifida. Reproduced with permission from Universitair Ziekenhuis Leuven, Belgium.

## References

[1] Khoshnood B , Loane M , de Walle H , Arriola L , Addor MC , Barisic I , et al. Long term trends in prevalence of neural tube defects in Europe: population based study. BMJ 2015;351:h5949. 26601850 10.1136/bmj.h5949 PMC4658393

[2] Bowman RM , Mclone DG , Grant JA , Tomita T , Ito JA . Spina bifida outcome: a 25‐year prospective. Pediatr Neurosurg 2001;34:114–20. 11359098 10.1159/000056005

[3] de Jong TP , Chrzan R , Klijn AJ , Dik P . Treatment of the neurogenic bladder in spina bifida. Pediatr Nephrol 2008;23:889–96. 18350321 10.1007/s00467-008-0780-7 PMC2335291

[4] Velde Vande S , Van Biervliet S , Van Renterghem K , Van Laecke E , Hoebeke P , Van Winckel M . Achieving fecal continence in patients with spina bifida: a descriptive cohort study. J Urol 2007;178:2640–4. 17945290 10.1016/j.juro.2007.07.060

[5] Verhoef M , Barf HA , Vroege JA , Post MW , Van Asbeck FW , Gooskens RH , et al. Sex education, relationships, and sexuality in young adults with spina bifida. Arch Phys Med Rehabil 2005;86:979–87. 15895345 10.1016/j.apmr.2004.10.042

[6] Teo C , Jones R . Management of hydrocephalus by endoscopic third ventriculostomy in patients with myelomeningocele. Pediatr Neurosurg 1996;25:57–63. 9075248 10.1159/000121098

[7] Roach JW , Short BF , Saltzman HM . Adult consequences of spina bifida: a cohort study. Clin Orthop Relat Res 2011;469:1246–52. 20878278 10.1007/s11999-010-1594-z PMC3069297

[8] Oakeshott P , Hung G , Poulton A , Reid F . Expectation of life and unexpected death in open spina bifida: a 40‐year complete, non‐selective, longitudinal cohort study. Dev Med Child Neurol 2010;52:749–53. 20015251 10.1111/j.1469-8749.2009.03543.x

[9] Vermaes IPR , Janssens JMAM , Bosman AMT , Gerris JRM . Parents’ psychological adjustment in families of children with spina bifida: a meta‐analysis. BMC Pediatr 2005;5:1–13. 16120229 10.1186/1471-2431-5-32 PMC1215488

[10] Bellin MH , Bentley KJ , Sawin KJ . Factors associated with the psychological and behavioral adjustment of siblings of youths with spina bifida. Fam Syst Health 2009;27:1–15. 19630441 10.1037/a0014859

[11] Shields N , Taylor NF , Dodd KJ . Self‐concept in children with spina bifida compared with typically developing children. Dev Med Child Neurol 2008;50:733–43. 18834386 10.1111/j.1469-8749.2008.03096.x

[12] Buran CF , Sawin KJ , Brei TJ , Fastenau PS . Adolescents with myelomeningocele: activities, beliefs, expectations, and perceptions. Dev Med Child Neurol 2004;46:244–52. 15077702 10.1017/s0012162204000404

[13] Wide K , Winbladh B , Kallen B . Major malformations in infants exposed to antiepileptic drugs in utero, with emphasis on carbamazepine and valproic acid: a nation‐wide, population‐based register study. Acta Paediatr 2004;93:174–6. 15046269 10.1080/08035250310021118

[14] Northrup H , Volcik KA . Spina bifida and other neural tube defects. Curr Probl Pediatr 2000;30:313–32. 11147289 10.1067/mpp.2000.112052

[15] Stothard KJ , Tennant PWG , Bell R , Rankin J . Maternal overweight and obesity and the risk of congenital anomalies: a systematic review and meta‐analysis. JAMA 2015;301:636–50. 10.1001/jama.2009.113 19211471

[16] Prevention of neural tube defects: results of the Medical Research Council Vitamin Study. MRC Vitamin Study Research Group. Lancet 1991;338:131–7. 1677062

[17] Bestwick JP , Huttly WJ , Morris JK , Wald NJ . Prevention of neural tube defects: a cross‐sectional study of the uptake of folic acid supplementation in nearly half a million women. PLoS One 2014;9:e89354. 24586711 10.1371/journal.pone.0089354 PMC3929694

[18] Kancherla V , Wagh K , Johnson Q , Oakley GP . A 2017 global update on folic acid‐preventable spina bifida and anencephaly. Birth Defects Res 2018;110:1139–47. 30070772 10.1002/bdr2.1366

[19] Morris JK , Rankin J , Draper ES , Kurinczuk JJ , Springett A , Tucker D , et al. Prevention of neural tube defects in the UK: a missed opportunity. Arch Dis Child 2016;101:604–7. 26681697 10.1136/archdischild-2015-309226 PMC4941168

[20] Boyd P , Wellesley D , De Walle H , Tenconi R , Garcia‐Minaur S , Zandwijken GR , et al. Evaluation of the prenatal diagnosis of neural tube defects by fetal ultrasonographic examination in different centres across Europe. J Med Screen 2000;7:169–74. 11202581 10.1136/jms.7.4.169

[21] Nadel A , Green J , Holmes L , Frigoletto F , Benacerraf B . Absence of need for amniocentesis inpatients with elevated levels of maternal serum alpha‐fetoprotein and normal ultrasonographic examinations. N Engl J Med 1990;323:557–61. 1696358 10.1056/NEJM199008303230901

[22] Public Health England . NHS Fetal Anomaly Screening Programme Handbook Valid from August 2018. London: Public Health England; 2018 [https://assets.publishing.service.gov.uk/government/uploads/system/uploads/attachment_data/file/749742/NHS_fetal_anomaly_screening_programme_handbook_FINAL1.2_18.10.18.pdf].

[23] Wald NJ , Cuckle H , Brock JH , Peto R , Polani PE , Woodford FP . Maternal serum‐alpha‐fetoprotein measurement in antenatal screening for anencephaly and spina bifida in early pregnancy. Report of UK collaborative study on alpha‐fetoprotein in relation to neural‐tube defects. Lancet 1977;1:1323–32. 69055

[24] Engels AC , Joyeux L , Brantner C , De Keersmaecker B , De Catte L , Baud D , et al. Sonographic detection of central nervous system defects in the first trimester of pregnancy. Prenat Diagn 2016;36:266–73. 26732542 10.1002/pd.4770

[25] Ushakov F , Sacco A , Andreeva E , Tudorache S , Everett T et al. Crash sign: a new first trimester sonographic marker of spina bifida. Ultrasound Obstet Gynecol 2019 10.1002/uog.20285. [Epub ahead of print] 30977215

[26] Ovaere C , Eggink A , Richter J , Cohen‐Overbeek TE , Van Calenbergh F , Jansen K , et al. Prenatal diagnosis and patient preferences in patients with neural tube defects around the advent of fetal surgery in Belgium and Holland. Fetal Diagn Ther 2015;37:226–34. 25301576 10.1159/000365214

[27] Tolcher M , Shazly S , Shamshirsaz A , Whitehead WE , Espinoza J , Vidaeff AC , et al. Neurological outcomes by mode of delivery for fetuses with open neural tube defects: a systematic review and meta‐analysis. BJOG 2018;1–6. 29924919 10.1111/1471-0528.15342

[28] Sival D , Begeer J , Staal‐Schreinemachers A . Perinatal motor behaviour and neurological outcome in spina bifida aperta. Early Hum Dev 1997;50:27–37. 9467691 10.1016/s0378-3782(97)00090-x

[29] Meuli M , Meuli‐Simmen C , Yingling CD , Hutchins GM , Hoffman KM , Harrison MR , et al. Creation of myelomeningocele in utero: a model of functional damage from spinal cord exposure in fetal sheep. J Pediatr Surg 1995;30:1028–32. 7472926 10.1016/0022-3468(95)90335-6

[30] Joyeux L , De Bie F , Danzer E , Van Mieghem T , Flake AW , Deprest J . Safety and efficacy of fetal surgery techniques to close a spina bifida defect in the fetal lamb model: a systematic review. Prenat Diagn 2018;38:231–42. 29388237 10.1002/pd.5222

[31] Adzick NS , Sutton LN , Crombleholme TM , Flake AW . Successful fetal surgery for spina bifida. Lancet 1998;352:1675–6. 9853442 10.1016/S0140-6736(98)00070-1

[32] Adzick NS , Thom EA , Spong CY , Brock JW 3rd , Burrows PK , Johnson MP , et al. A randomized trial of prenatal versus postnatal repair of myelomeningocele. N Engl J Med 2011;364:993–1004. 21306277 10.1056/NEJMoa1014379 PMC3770179

[33] Tulipan N , Wellons JC 3rd , Thom EA , Gupta N , Sutton LN , Burrows PK , et al. Prenatal surgery for myelomeningocele and the need for cerebrospinal fluid shunt placement. J Neurosurg Pediatr 2015;16:613–20. 26369371 10.3171/2015.7.PEDS15336 PMC5206797

[34] Johnson MP , Bennett KA , Rand L , Burrows PK , Thom EA , Howell LJ , et al. The Management of Myelomeningocele Study: obstetrical outcomes and risk factors for obstetrical complications following prenatal surgery. Am J Obstet Gynecol 2016;215:778.e1–778.e9. 27496687 10.1016/j.ajog.2016.07.052 PMC5896767

[35] Farmer DL , Thom EA , Brock JW 3rd , Burrows PK , Johnson MP , Howell LJ , et al. The Management of Myelomeningocele Study: full cohort 30‐month pediatric outcomes. Am J Obstet Gynecol 2017;218:256.e1–256.e13. 29246577 10.1016/j.ajog.2017.12.001 PMC7737375

[36] Soni S , Moldenhauer JS , Spinner SS , Rendon N , Khalek N , Martinez‐Poyer J , et al. Chorioamniotic membrane separation and preterm premature rupture of membranes complicating in utero myelomeningocele repair. Am J Obstet Gynecol 2016;214:647.e1–7. 26692177 10.1016/j.ajog.2015.12.003

[37] Danzer E , Gerdes M , Bebbington MW , Zarnow DM , Adzick NS , Johnson MP . Preschool neurodevelopmental outcome of children following fetal myelomeningocele closure. Am J Obstet Gynecol 2010;202:450.e1–9. 20347433 10.1016/j.ajog.2010.02.014

[38] Danzer E , Gerdes M , Bebbington MW , Koh J , Adzick SN , Johnson MP . Fetal myelomeningocele surgery: preschool functional status using the Functional Independence Measure for children (WeeFIM). Childs Nerv Syst 2011;27:1083–8. 21327591 10.1007/s00381-011-1388-y

[39] Danzer E , Gerdes M , Bebbington MW , Koh J , Adzick NS , Johnson MP . Preschool neurobehavioral outcome following fetal myelomeningocele surgery. Fetal Diagn Ther 2011;30:174–9. 21912086 10.1159/000330048

[40] Danzer E , Thomas NH , Thomas A , Friedman KB , Gerdes M , Koh J , et al. Long‐term neurofunctional outcome, executive functioning, and behavioral adaptive skills following fetal myelomeningocele surgery. Am J Obstet Gynecol 2016;214:269 e1–269.e8. 26440692 10.1016/j.ajog.2015.09.094

[41] Sacco A , Van der Veeken L , Bagshaw E , Ferguson C , Van Mieghem T , David AL , et al. Maternal complications following open and fetoscopic fetal surgery: a systematic review and meta‐analysis. Prenat Diagn 2019;39:251–68. 30703262 10.1002/pd.5421 PMC6492015

[42] Deprest J , Toelen J , Debyser Z , Rodrigues C , Devlieger R , De Catte L , et al. The fetal patient – ethical aspects of fetal therapy. Facts Views Vis Obgyn 2011;3:221–7. 24753868 PMC3991449

[43] Van Calenbergh F , Joyeux L , Deprest J . Maternal–fetal surgery for myelomeningocele: some thoughts on ethical, legal and psychological issues in a Western European situation. Childs Nerv Syst 2017;33:1247–52. 28536839 10.1007/s00381-017-3446-6

[44] Werner E , Han C , Burd I . Evaluating the cost‐effectiveness of prenatal surgery for myelomeningocele: a decision analysis. Ultrasound Obs Gynecol 2012;40:158–64. 10.1002/uog.11176 22511529

[45] Sacco A , Simpson L , Deprest J , David AL . A study to assess global availability of fetal surgery for myelomeningocele. Prenat Diagn 2018;38:1020–7. 30378145 10.1002/pd.5383 PMC7613409

[46] Botelho R , Imada V , Rodrigues da Costa K , Watanabe L , Rossi Júnior R , De Salles AAF , et al. Fetal myelomeningocele repair through a mini‐hysterotomy. Fetal Diagn Ther 2017;42:28–34. 27656888 10.1159/000449382

[47] Kabagambe S , Jensen G , Chen YJ , Vanover MA , Farmer DL . Fetal surgery for myelomeingocele: a systematic review and meta‐analysis of outcomes in fetoscopic versus open repair. Fetal Diagn Ther 2018;43:161–74. 28910784 10.1159/000479505

[48] Skinner S , Dekoninck P , Crossley K , Amberg B , Deprest J , Hooper S , et al. Partial amniotic carbon dioxide insufflation for fetal surgery. Prenat Diagn 2018;38:983–93. 30238473 10.1002/pd.5362

[49] Joyeux L , De Bie F , Danzer E , Russo FM , Javaux A et al. (2019), Learning curves of open and endoscopic fetal spina bifida closure: a systematic review and meta‐analysis. Ultrasound Obstet Gynecol. (In press). 10.1002/uog.20389 31273862

[50] American College of Obstetricians and Gynecologists . ACOG Committee opinion no. 720: maternal–fetal surgery for myelomeningocele. Obstet Gynecol 2017;130:e164–7. 28832491 10.1097/AOG.0000000000002303

[51] GIFTSurg project [https://www.gift-surg.ac.uk].

