# Supplementary material for: Fetal surgery for open spina bifida
Source: Obstet Gynaecol. 2019 Sep 27;21(4):271–82. doi: 10.1111/tog.12603 (PMC6876677; doi:10.1111/tog.12603)
Supplement: Supplementary file 2 [file TOG-21-271-s002.docx]

**Supporting Information 1:** Video of open fetal surgery for spina bifida. Reproduced with permission

from Universitair Ziekenhuis Leuven, Belgium.
